# Comparison of Financial Hardship and Healthcare Utilizations Associated with Cancer in the United States Medicare Programs during the COVID-19 Pandemic

**DOI:** 10.3390/healthcare12101049

**Published:** 2024-05-20

**Authors:** Jiamin Hu, Mishal Khan, Xiaobei Chen, Lee Revere, Young-Rock Hong

**Affiliations:** 1 Department of Health Services Research, Management and Policy, College of Public Health and Health Professions, University of Florida, Gainesville, FL 32611, USA; jiaminhu@phhp.ufl.edu (J.H.); mkhan2321@phhp.ufl.edu (M.K.); frevere@phhp.ufl.edu (L.R.); 2 College of Journalism and Communications, University of Florida, Gainesville, FL 32611, USA; xiaobei.chen@ufl.edu

**Keywords:** Medicare Advantage, cancer survivorship, financial burden, healthcare utilization

## Abstract

Background: In the United States, Medicare beneficiaries diagnosed with cancer often face significant financial challenges due to the expensive nature of cancer treatments and increased cost-sharing responsibilities. However, there is limited knowledge regarding the financial hardships and healthcare utilizations faced by those enrolled in Medicare Advantage (MA) compared to those in traditional fee-for-service Medicare (TM) during the COVID-19 pandemic. Our study aims to investigate the subjective financial hardships experienced by individuals enrolled in TM and MA and to determine whether these two Medicare programs exhibit differences in healthcare utilization during the pandemic. Methods: We utilized data from the 2020–2022 National Health Interview Survey (NHIS), focusing on nationally representative samples of cancer survivors aged 65 or older. Financial hardship was categorized into three distinct groups: material (e.g., problems with medical bills), psychological (e.g., worry about paying), and behavioral (e.g., delayed care due to cost). Healthcare utilization included wellness visits (preventive care), emergency care services, hospitalizations, and telehealth. We used survey design-adjusted analysis to compare the study outcomes between MA and TM. Results: Among a weighted sample of 4.4 million Medicare beneficiaries with cancer (mean age: 74.9), 76% were enrolled in MA plans. Cancer survivors with a college degree (59.3% vs. 49.8%) and high family income (38.2% vs. 31.1%) were more likely to enroll in MA plans. There were no significant differences in any material, psychological, or behavioral financial hardship domains between beneficiaries with MA and TM plans except forgone counseling due to cost. For healthcare utilization measures, cancer survivors in MA were more likely than those in TM to have flu vaccination (77.2% vs. 70.1%) and experience lower hospitalizations (16.0% vs. 20.0%). However, there were no differences in other health service utilizations between MA and TM. Conclusion: While no significant differences were observed in any materialized, psychological, or behavioral financial hardships, older cancer survivors enrolled in MA plans were more likely to receive vaccinations and lower hospitalization rates during COVID-19. Although other preventive or primary care visits (i.e., wellness visits) were higher, their difference did not reach statistical significance. As MA grows in popularity, it is essential to consistently monitor and evaluate the performance and outcomes of Medicare plans for cancer survivors as we navigate the post-pandemic landscape.

## 1. Introduction

Cancer has affected over 18 million Americans since 2022 and had an estimated economic burden of USD 21 billion in 2019, directly impacting their well-being [1,2]. In the United States (US), annual cancer-related expenses ranked highest for senior cancer patients when compared to other age groups, both at the initial stage and the end-of-life phases [2]. For cancer survivors aged 65 years or older, annual out-of-pocket costs for medical services and prescription drugs exceeded USD 2200 (medical) and USD 243 (prescription) at the initial cancer stage, and USD 3823 (medical) and USD 448 (prescription) at the end-of-life phase [1,2]. The burden of cancer-related expenses can be particularly daunting, given the costly nature of treatments and the increasing responsibilities of out-of-pocket payments. With the growth in the US aging population and the increased likelihood of a cancer diagnosis, there may be a substantial financial burden for both cancer patients and their health insurers in the coming years [3,4]. The material, psychological, and behavioral are three financial challenges cancer patients may experience during their survivorship [5]. Previous research has defined material hardship as difficulties in paying medical bills, psychological hardships as worrying about paying medical bills, and behavioral hardships as delaying and/or forgoing care, prescriptions, and counseling [4,6].

Health insurance, especially public insurance programs such as Medicare, Medicaid, and Veterans Health Administration (VHA) or military coverage, is designed to provide individuals with greater healthcare access and reduce costs compared to employer-sponsored or private insurance programs [7]. Medicare, a federal health insurance program, comprises traditional Medicare (TM) and Medicare Advantage (MA), primarily catering to individuals aged 65 and older and plays a crucial role in ensuring access to necessary medical services [8]. Given that over 75% of cancer survivors are adults aged 60 years and older, many of whom are Medicare-eligible, they often face the complex decision of choosing between TM and MA [1]. MA plans, operated by private insurance companies, cover over 50% of all Medicare-eligible beneficiaries (31 million) as of 2023 [9,10]. MA enrollees can choose from various private insurers, each offering its own set of benefits and limitations. For example, most MA plans provide covered or extended benefits beyond those offered by TM, such as network flexibility, dental care, or fitness memberships [11,12,13], and lower out-of-pocket expenses, which is a primary reason many seniors select an MA plan [9,13]. In addition, MA plans often outperform TM at coordinating or managing chronic health conditions, as they are financially incentivized to maintain cost [14]. The level of financial hardships is one of the considerations when people choose an insurance plan, a research gap exists in defining financial hardships among cancer survivors between MA plans versus TM.

Healthcare utilization is another consideration in evaluating the difference between MA plans and TM [15,16,17,18]. Research indicates that MA plans are more effective in reducing costs, decreasing hospitalizations, and raising access to healthcare utilization, including primary care, annual health checks, and preventive care services such as vaccinations [15,19,20,21]. However, individuals in an MA plan may encounter limitations, such as being restricted from using providers and facilities within the MA’s network and needing referrals for most specialty and hospital services [11]. Additionally, MA plans require patients to use contracted providers, potentially restricting cancer patients from receiving services at top-rank hospitals or from the best disease experts who are not in the MA plan network [1,22].

The COVID-19 pandemic also worsens the overall access to healthcare utilization and preventive care services, largely due to patients delaying or forgoing necessary care, or annual checks across all health plans, including TM and MA plans [23,24]. The reduction in utilization has resulted in a negative health impact on the general population [23]. Although telehealth was proven to increase some access to healthcare during the COVID-19 pandemic, evidence shows the pandemic’s negative health consequences continue to influence healthcare utilization, and utilization rates have not rebounded to the pre-pandemic levels [24,25,26].

Overall, to our knowledge, little is known about whether differences exist in both healthcare utilization and financial hardships between TM and MA plans, specifically among cancer survivors who have higher rates of comorbid conditions, economic concerns, and a need for primary care [2,17,27,28]. While both TM and MA plans offer similar healthcare services, even during the pandemic, MA plans often require referrals and approvals, which may extend waiting times or be denied [11,17]. The COVID-19 pandemic has impacted every aspect of healthcare; therefore, it is imperative to conduct studies to examine the challenges and potential disparities in healthcare service utilization and associated financial hardship that may have emerged during this unprecedented time.

Our study aims to address this gap by investigating the subjective financial hardships experienced by individuals enrolled in TM and MA, and whether those two Medicare programs differ in healthcare utilization. We utilize comprehensive data from the 2020 to 2022 National Health Interview Survey (NHIS), focusing on nationally representative samples of cancer survivors aged 65 or older during the COVID-19 pandemic. Our research delves into the intricate facets of financial hardship by categorizing them into three distinct groups: material difficulties, such as struggling to pay overwhelming medical bills; psychological concerns, such as anxiety about settling medical debts; and behavioral adaptations, such as postponing or abstaining from necessary care due to financial constraints. Additionally, we studied information on healthcare utilization in the NHIS dataset, including wellness visits, emergency services, flu vaccination, hospitalization history, and telehealth. Understanding differences in financial hardships and healthcare utilization among cancer survivors in MA vs. TM is important as policymakers evaluate ways to improve health equity, especially considering that MA beneficiaries constitute over 50 percent of Medicare enrollees.

## 2. Methods

### 2.1. Data and Study Population

This cross-sectional study included Medicare enrollees diagnosed with cancer who responded to the 2020 to 2022 NHIS. The NHIS is an annual survey conducted by the National Center for Health Statistics, aimed at monitoring the health status of the civilian noninstitutionalized US population and providing a nationally representative sample each year. The adult sample for this survey was randomly chosen from all 50 states and the District of Columbia for further interviews (2020, N = 31,568; 2021, N = 29,482; 2022, N = 27,651). NHIS provided questionnaire variables to determine health insurance status. Our research included individuals aged 65 and over who were enrolled in TM or MA plans and excluded those with private, dual-eligible, or other coverage populations. We also excluded samples that self-reported non-melanoma skin cancer or were uncertain about their skin cancer type. Cancer survivors were defined as individuals informed by a physician or other healthcare provider that they had cancer or any other type of malignancy. We identified 2202 cancer survivors aged 65 and over who were TM or MA enrollees, resulting in a weighted cohort of 4,387,278 sample adults, of whom 1,047,759 (24%) were enrolled in TM and 3,339,519 (76%) were enrolled in MA. Since the data used in this study were fully deidentified and publicly available, the Institutional Review Board of the University of Florida determined that the study was exempt from review and the requirement for informed consent.

### 2.2. Outcomes

We categorized financial hardships associated with cancer, treatment, or the lasting effects of treatment into three domains: (1) material, (2) psychological, and (3) behavioral [29]. Material-financial hardship measures included problems paying medical bills and being unable to pay medical bills within the past 12 months. Psychological-financial hardship measures included worrying about paying medical bills if you become sick or have an accident. Behavioral-financial hardships were defined as reporting delaying or forgoing medical care, prescription medication, or counseling due to cost during the past 12 months. We created two composite measures for financial burden outcomes (yes/no) for reporting material or behavioral measures. Any material financial hardship was defined as answering ‘yes’ to one or more of the individual material financial hardship measures, including problems paying medical bills and/or being unable to pay medical bills. Any behavioral financial hardship was defined as answering ‘yes’ to one or more of the adherence measures, including having delayed and/or forgone care due to cost, delayed and/or forgone prescription medication, and/or delayed and/or forgone prescription counseling due to cost. Since there is only one question collected within the 2020–2022 NHIS dataset for psychological hardship, no composite outcomes were created under this section.

Healthcare utilization was collected from the NHIS questionnaire, including wellness visits, emergency services, flu vaccination, hospitalization, and telehealth utilization within the past 12 months. Wellness visits included checks for blood pressure, cholesterol, blood sugar, and height and weight measurements. Emergency services included urgent care and emergency department (ED) visits.

### 2.3. Covariates

Baseline characteristic variables included age reported at each year of the survey, race and ethnicity (non-Hispanic White, Hispanic, non-Hispanic Black, or other), sex, education levels, marital status, geographic region, number of comorbid conditions (0, 1, 2, 3, or ≥3), cancer site (breast, prostate, skin melanoma, multiple cancers, lung cancer, all other cancer sites), and family income level as a percentage of the federal poverty level (FPL) (<200%, 200–399%, or ≥400%). Other races/ethnicities include non-Hispanic Asian only, non-Hispanic American Indian or Alaska Native (AIAN) only, non-Hispanic AIAN and any other group, or those who identify with other single and multiple races. Comorbid conditions included self-reported arthritis, asthma, diabetes, chronic obstructive pulmonary disease (COPD), coronary heart disease, hypertension, stroke, angina pectoris, and heart attack. Multiple cancers refer to having more than one cancer site. All other cancer sites were grouped for all lower diagnosed cancer sites, including bladder, blood, bone, brain, cervical, colon, esophageal, gallbladder, larynx trachea, leukemia, liver, uterine, lymphoma, melanoma, mouth, ovarian, pancreatic, rectal, stomach, throat, thyroid, head & neck, colorectal, and other cancer.

### 2.4. Statistical Analysis

We compared the distribution of baseline cancer survivors between TM and MA using the Chi-square test with Rao and Scott’s second-order correction in complex survey samples. We utilized several survey design-weighted logistic regression models to compare financial hardship and healthcare utilization outcomes among Medicare beneficiaries enrolled in TM and MA. These models were adjusted for survey year, age, sex, race, education level, marital status, region, number of comorbid conditions, and family income level as a percentage of the federal poverty level. For the main analysis, we opted not to stratify or adjust for specific cancer types due to limited sample sizes for certain cancer subtypes, which could introduce statistical power issues and reduce the reliability of the results. This approach was taken to enhance the generalizability of our findings and to provide a broader understanding of how different Medicare program types are associated with study outcomes for all cancer survivors. We treated missing data with listwise deletion. For the study outcome, including questions related to financial hardships or healthcare utilizations, all missing data were retained. All estimates were weighted to represent the national population using NHIS complex survey design weights, and analyses were conducted using R studio software (R version 4.3.1). Statistical significance was determined with a 2-sided *p* < 0.05.

## 3. Results

The weighted study samples included 4,387,278 elderly Medicare beneficiaries with a history of cancer, among whom 1,047,759 (24%) were TM enrollees and 3,339,519 (76%) were MA enrollees. The average age of the participants was 74.9 years (95% CI, 74.6–75.3). An analysis revealed a statistically significant association between the selection of TM or MA plans and factors such as education levels and family income level as a percentage of the federal poverty level (Table 1). Beneficiaries with at least a college degree were more likely to enroll in MA plans than those without less than high school (59.3% vs. 49.8%; *p* < 0.001). The family income level as a percentage of the federal poverty level less than 200% were more likely to opt for TM plans (<200%, 27.9% vs. 38.4%; 200%–399% 33.9% vs. 30.5%; ≥400% 38.2% vs. 31.1%; *p* < 0.001).

Between the TM and MA groups, other baseline characteristics like race and entity (non-Hispanic White, 82.0% vs. 77.9%; Hispanic, 6.4% vs. 5.8%; non-Hispanic Black, 7.6% vs. 10.9%; Other, 4.0% vs. 5.4%), sex (male, 40.9% vs. 39.0%; female, 59.1% vs. 61.0%), marital status (married, 56.3% vs. 55.4%; not married, 43.7% vs. 44.6%), the number of comorbid conditions (0, 11.4% vs. 12.5%; 1, 30.1% vs. 28.5%; 2, 27.9% 27.1%; ≥3, 30.6% vs. 32.0%), region (West, 24.8% vs. 21.5%; Midwest, 20.5% vs. 17.5%; South, 37.9% vs. 39.7%; Northeast, 16.8% vs. 21.2%), and cancer sites (breast, 23.1% vs. 24.3%; prostate, 15.1% vs. 15.9%; skin melanoma, 10.2% vs. 7.1%; multiple cancers, 10.5% vs. 7.5%; lung, 3.5% vs. 2.6%; all other, 37.6% vs. 42.6%;) had weak associations with choices of TM or MA plans.

### 3.1. Financial Hardships

For measurements of material financial hardships, we observed no significant differences in problems of paying medical bills (11.8% in TM vs. 10.3% in MA; aOR [95% CI, 0.7, 1.55]) or being unable to pay medical bills (6.2% in TM vs. 5.7% in MA; aOR [95% CI, 0.5, 2.47]) among adults who enrolled in TM or MA plans (Table 2).

Psychological concerns like worrying about paying medical bills if you become sick or have an accident were not associated with financial hardships for cancer TM and MA enrollees in our survey (38.2% in TM vs. 33.0% in MA; aOR [95% CI, 0.73, 1.2]).

Adults aged 65 years and over who are diagnosed with cancer and in MA are slightly less likely to have behavioral financial hardship in delaying medical care (4.8% in TM vs. 3.0% in MA; aOR [95% CI, 0.37, 1.31]), prescription medication (3.5% in TM vs. 3.6% in MA; aOR [95% CI, 0.61, 2.03]), and counseling due to cost (1.4% in TM vs. 0.7% in MA; aOR [95% CI, 0.17, 1.11]) during the past 12 months as well as in forgoing medical care (4.6% in TM vs. 3.3% in MA; aOR [95% CI, 0.48, 1.67]) or prescription medications (6.2% in TM vs. 4.3% in MA; aOR [95% CI, 0.44, 1.40]) during the past 12 months. However, we found that cancer survivors aged 65 years and older in MA plans were more likely to have forgone counseling due to cost (2.2% in TM vs. 1.0% in MA; aOR [95% CI, 0.16, 0.92], *p* = 0.03) during the past 12 months.

Among those cancer survivors aged 65 years and over, we did not observe any significant difference between TM or MA plans to report any material (11.8% in TM vs. 10.3% in MA; aOR [95% CI, 0.64, 1.43]) or behavioral financial (12.0% in TM vs. 8.7% in MA; aOR [95% CI, 0.86, 1.91]) hardships.

### 3.2. Healthcare Utilization

Cancer survivors enrolled in MA plans are more likely to receive a flu vaccination (70.1% in TM vs. 77.2% in MA; aOR [95% CI, 1.03, 1.67], *p* = 0.03) and have a lower rate of hospitalization (20.1% in TM vs. 16% in MA; aOR [95% CI, 0.56, 0.98], *p* = 0.03) than those in TM plans. The distribution between TM and MA plans in any wellness visit plans (14.8% in TM vs. 16.8% in MA; aOR [95% CI, 0.63, 3.14]), emergency services (39.9% in TM vs. 39.6% in MA; aOR [95% CI, 0.83, 1.35]), and telehealth (33.4% in TM vs. 37.9% in MA; aOR [95% CI, 0.67, 1.14]) were similar among cancer Medicare beneficiaries, and no significant differences were observed (Table 3).

## 4. Discussion

The aim of this study was to investigate self-reported financial hardship and healthcare services utilization among cancer patients during the COVID-19 pandemic, comparing Medicare Advantage (MA) and Traditional Medicare (TM) plans. Overall, our findings revealed a few significant differences between MA and TM plans regarding financial hardship and healthcare services usage among cancer survivors aged 65 and over. MA enrollees were more likely to receive flu vaccinations and less likely to miss counseling due to cost, as well as having lower hospitalization rates compared to TM enrollees. However, both plans exhibited a similar proportion of older cancer survivors utilizing emergency services, wellness visits, and telehealth services. Additionally, we found no significant differences in any material, psychological, or behavioral hardships between elderly TM and MA enrollees with a history of cancer after the COVID-19 pandemic. Below, we discuss the implications of our study findings.

One key difference between MA and TM is that MA plans have had a legally defined maximum out-of-pocket limit since 2011, whereas TM does not [9]. In 2023, Medicare Advantage plans provided an out-of-pocket (OOP) maximum of USD 8300 for in-network services to enrollees [9]. This out-of-pocket cap in MA offers financial protection and limits catastrophic spending for cancer patients undergoing expensive treatments. Thus, we expected TM enrollees to be more likely to experience financial hardships because MA plans offer financial protection, which limits the enrollee’s cost for high spending typical of cancer patients undergoing expensive treatments. Previous research indicates that cancer patients are at high risk for financial toxicity due to increasingly expensive cancer care and significant cost-sharing [30,31,32,33]. This finding suggests that despite the out-of-pocket limit, MA beneficiaries with a history of cancer still face substantial financial burdens comparable to those under TM.

Our study did not find significantly lower financial hardship in the MA group compared to TM. One possible explanation for the lack of financial hardship difference between TM and MA enrollees is the prevalence of Medicare Parts B and D (a supplement available for purchase) for TM enrollees [34]. In 2021, over 80% of TM enrollees had supplemental coverage, such as Medicare supplements, employer coverage, and Medicaid, which may lead to less economic burden than MA enrollees [8,35]. Medicare Part B offers a reprieve from some expenses by reducing the out-of-pocket (OOP) expenses for TM enrollees. For example, TM enrollees with a Medicare Part B supplement have a USD 1632 deductible per benefit period and a small co-insurance for hospital stays (e.g., Days 0–60: USD 0, Days 61–90: USD 408 per Day, Days 91–150: USD 816 per Day by Lifetime Reserve Days) [36]. Another potential hypothesis is that out-of-pocket costs for TM cancer survivors, including deductibles and co-insurance, do not exceed the MA plan’s max OOP of USD 8300. Therefore, we may not observe any significant difference in medical OOP expenditure between TM and MA plans. Similarly, many TM beneficiaries have Parts B and D, which cover physician and medication benefits, and the Medicare Part D supplement reduces OOP expenses associated with medications [34,37]. Thus, it is reasonable to expect a narrow gap between the TM and MA plans, as indicated by our results. These findings are consistent with previous studies that have demonstrated Medicare beneficiaries with cancer are susceptible to financial hardship due to the increasing costs of cancer care and substantial cost-sharing, regardless of their Medicare plan type [38].

Another possible interpretation is that additional insurance coverage reduces the gap between TM and MA beneficiaries. Medigap coverage prevents catastrophic medical expenses and limits the financial exposure of TM beneficiaries [8]. TM enrollees with employer or union coverage are likelier to have higher income, educational level, or better health conditions, suggesting they should face fewer medical bills [8]. Medicaid, a federal–state insurance program designed to assist low-income populations, also plays a role in reducing financial strain [8]. Dual-eligible beneficiaries with TM and Medicaid can have additional cost-sharing benefits or long-term services that share some financial and health service responsibilities [8]. Based on our findings, there was no significant difference in financial hardships or healthcare utilization between the TM and MA plans. Thus, it is possible that supplemental coverage may have helped reduce the financial strain of healthcare expenses and increased access to care during the pandemic. By covering some or all the out-of-pocket costs, cancer survivors are more likely to seek the care they need without stressing about the financial impact.

Our study sheds light on this matter by highlighting a higher number of wellness visits, similar emergency service utilization, significant differences and high access to flu vaccinations, lower hospitalizations, and greater telehealth usage with MA plans compared to those with TM plans. For the general population, MA plans are financially incentivized to coordinate beneficiaries’ care. Research has consistently demonstrated that MA enrollees are more likely to undergo more frequent preventive care visits, have fewer emergency department visits, and experience fewer hospital admissions [18,39,40]. However, existing studies have provided little evidence regarding differences in health service utilization between MA and TM among cancer subgroups and/or following the COVID-19 pandemic [4,6,17,26,41,42]. Since 2020, MA plans have been able to offer the same telehealth benefits as TM plans for all enrollees, which could partially explain why more MA beneficiaries than TM beneficiaries utilized telehealth in our study [43]. Additionally, lower healthcare utilization associated with telehealth’s efficiency and high quality has been evidenced by reduced hospital bed days or emergency department visits [44]. Furthermore, the increasing use of telehealth during the pandemic provided Medicare enrollees with convenience and lower cost [45]. Telehealth served as a substitute for in-person healthcare services, such as wellness visits, which aligns with our study results indicating a lower proportion of MA plan enrollees reporting material, psychological, and behavioral financial hardships compared to TM plans, possibly due to higher telehealth utilization [26].

On the other hand, previous research has further indicated that cancer patients, especially the elderly, experienced higher rates of hospital admission and emergency department visits than the general population [46,47]. Nevertheless, MA programs provide patients with lower rates of hospitalization and emergency care utilization, even though cancer patients typically require emergency care and hospitalization more frequently than the general population. The high use of telemedicine and low healthcare utilization offset each other, which explains the lack of differences between TM and MA in our study.

With respect to care management, MA plans are financially incentivized through payment structures and the Quality Bonus Program [48] to manage and coordinate beneficiaries’ care. However, concerns have arisen regarding whether such financial incentive programs actually improve the quality of care or result in lower medical expenditures for both enrollees and payers [9,48,49]. There is no doubt that the MA plan will try its best to make sure their enrollees achieve more access to healthcare utilization like wellness visits to meet financial credits [48]. The MA plans can also be called managed care, which aims to avoid unnecessary care [50]; for example, bring patients out to the hospital as quickly as possible and do everything possible to make sure they are not readmitted by scheduling lots of follow-up care and coordinating primary care physician (PCP) visits. Those common rules are consistent with our finding that the MA plan is accountable for a higher rate of wellness visits or a lower rate of ED visits and hospital use. We can also conclude that even if MA can contain costs better than TM for unnecessary healthcare services [20], the health outcomes for both TM and MA are proven to be good and similar in our research.

The impact of racial and ethnic disparities and socioeconomic status (SES) on MA and TM plans has yielded conflicting results in previous studies, particularly regarding outcomes such as hospital readmission [51,52]. The racial and ethnic distribution of MA and TM enrollment varies by disease. For instance, in cases of diabetes and Alzheimer’s disease and related dementias (ADRDs), White individuals are more likely to enroll in TM plans, whereas MA tends to cover more Black diabetes patients, although this situation was limited to ADRDs [15,53]. However, our study finds that cancer survivors do not observe significant differences between the racial groups, which is consistent with other research regarding diseased-related subgroups of heart failure [54].

Another interesting finding in our study is that beneficiaries who have a higher SES like people who earned at least a college degree and family income above 200% are more likely to enroll in MA plans. This finding is inconsistent with the MA plan enrollments in the general population [8,55]; however, the same characteristics were found in ADRDs and diabetes [15,53]. This discrepancy may indeed be attributed to the marketing strategies employed by MA plans. A survey showed that obtaining information from advertising was more frequently utilized by elderly, low-income, and Black Medicare beneficiaries [56,57]. However, when confronted with numerous and possibly even false marketing, many seniors were not influenced to review the insurance plans they had chosen [57,58]. In addition, the content of MA’s advertisements routinely shows healthy older patients participating in physical activity, whereas such advertisements would potentially turn away patients with serious health problems [59]. Thus, we may expect that, unlike the general beneficiaries, unhealthy populations such as cancer survivors are less influenced by marketing or not the targeted population for the MA plans.

The COVID-19 pandemic had a limited impact on healthcare accessibility disparities across racial and ethnic groups, with fewer discrepancies observed in wellness visits and delayed visits due to cost. This trend could be attributed to the increased utilization of telemedicine during the pandemic [26]. Our study did not research the financial hardships and healthcare utilization for any racial and ethnic subgroups due to the small size. However, as MA plans continue to grow in popularity, representing over 50% of Medicare enrollees, it is essential for future studies to closely examine the experiences and outcomes of minority and low-income cancer patients in these Medicare plans [10]. Evidence on disparities and challenges can inform tailored policy solutions and educational interventions to enhance MA cancer care and reduce financial toxicity for diverse patients.

This study has several limitations. First, cancer diagnosis, health insurance status information, financial hardships, and healthcare utilization measures come from self-reported NHIS data. Using self-reported survey data might lead to recall bias and the misclassification of experiences within data analysis. Another significant limitation of the NHIS data is the absence of comprehensive information on medical expenses incurred by cancer survivors. Consequently, we relied solely on self-reported financial burden measures, which may be influenced by various factors beyond the scope of the survey. These factors could include the availability of supplemental insurance coverage, out-of-pocket costs for non-covered services or treatments, lost income due to the inability to work during treatment, and other financial strains associated with managing other chronic conditions [29,60,61]. Third, the sample size of individuals with cancer for each year or by cancer type is small, affecting the statistical power and generalizability of the findings. Although the NHIS sample is representative, due to the small size, certain characteristics or financial hardship groups could be underrepresented. Fourth, NHIS data are from an observational cross-sectional analysis, making it challenging to establish causal relationships. While associations can be identified, it is difficult to determine whether the observed differences between MA and TM programs are solely attributable to the type of insurance or influenced by other unobserved factors. Fifth, NHIS excludes individuals living in institutionalized settings, such as nursing homes or prisons. This exclusion may limit the generalizability of findings, especially for populations with unique healthcare needs like elderly cancer patients. Sixth, measuring complex financial hardship is inherently challenging, and the NHIS questions may not fully capture the multidimensional nature and nuances of individuals’ financial challenges. The questionnaire collected information from respondents and their family members for material financial hardship measures. Hence, our results of problems with paying or the inability to pay medical bills may not be attributed to cancer survivors themselves but may indicate that cancer survivors and their families are more likely to face financial issues. Additionally, long-term cancer patients might have a lower chance of receiving treatments during the survey year, so associated medical bills collected in the NIHS may not be clinically accurate. Lastly, we pooled data from 2020 to 2022; not all healthcare services were asked yearly. Thus, we only included variables collected all four years, which could underestimate healthcare utilization. Wellness visits consider the vaccination status and may include questions about flu vaccinations, leading to double counting in our statistical methods. 

## 5. Conclusions

While no significant differences were observed in any material, psychological, or behavioral financial hardships, older cancer survivors enrolled in MA plans were more likely to receive preventive care, such as vaccinations, were less likely to miss counseling due to cost, and experienced fewer hospitalizations during COVID-19. Although other preventive or primary care visits (i.e., wellness visits) were higher, the difference did not reach statistical significance. Overall, study findings suggest that during the COVID-19 pandemic, older cancer survivors’ access to care and the financial burdens they experienced were not significantly influenced by whether they were enrolled in MA or TM. As MA grows in popularity, it is important to consistently monitor and evaluate the performance and outcomes of Medicare plans for cancer survivors in the post-pandemic era.

## Figures and Tables

**Table 1:** Baseline characteristics for Medicare beneficiaries aged 65 years or older with cancer diagnoses.

Characteristic	Beneficiaries, % (95% CI)				
	Traditional Medicare (24%) ^1,2^		Medicare Advantage (76%) ^1,2^		
	n n = 532	Weighted % N = 1,047,759	n n = 1670	Weighted % N = 3,339,519	*p*-Value ^3^
Year					
2020	188	31.8% (27.3%, 36.7%)	566	31.2% (28.5%, 34.0%)	0.97
2021	172	34.1% (29.7%, 38.9%)	561	34.1% (31.4%, 37.0%)	
2022	172	34.1% (29.5%, 39.0%)	543	34.6% (32.0%, 37.4%)	
Age	75.3	74.7 (74.02, 75.32)	75.4	75.0 (74.65, 75.39)	0.33
Race					
Non-Hispanic White	427	77.9% (73.0%, 82.2%)	1424	82.0% (79.3%, 84.3%)	0.19
Hispanic	26	5.8% (3.80%, 8.7%)	76	6.4% (4.8%, 8.6%)	
Non-Hispanic Black	57	10.9% (8.1%, 14.5%)	117	7.6% (6.2%, 9.4%)	
Other ^4^	22	5.4% (3.1%, 9.2%)	53	4.0% (2.9%, 5.4%)	
Sex					
Male	191	39.0% (34.4%, 43.9%)	644	40.9% (38.3%, 43.6%)	0.51
Female	341	61.0% (56.1%, 65.6%)	1026	59.1% (56.4%, 61.7%)	
Education					
<High school	92	20.3% (16.2%, 25.2%)	171	12.7% (10.7%, 14.9%)	**<0.001**
High school graduate	151	29.9% (25.4%, 34.9%)	408	28.1% (25.4%, 30.9%)	
≥Some college	289	49.8% (44.9%, 54.6%)	1091	59.3% (56.3%, 62.2%)	
Marital					
Married	226	55.4% (50.3%, 60.4%)	736	56.3% (53.5%, 59.2%)	0.75
Not married	306	44.6% (39.6%, 49.7%)	934	43.7% (40.8%, 46.5%)	
Region					
Northeast	119	21.2% (16.7%, 26.5%)	266	16.8% (14.8%, 19.0%)	0.18
Midwest	101	17.5% (14.1%, 21.6%)	354	20.5% (18.2%, 22.9%)	
South	209	39.7% (34.6%, 45.1%)	612	37.9% (34.7%, 41.2%)	
West	103	21.5% (17.1%, 26.7%)	438	24.8% (22.0%, 27.8%)	
No comorbid conditions ^5^					
0	72	12.5% (9.68%, 15.9%)	196	11.4% (9.8%, 13.1%)	0.85
1	154	28.5% (24.0%, 33.4%)	498	30.1% (27.6%, 32.7%)	
2	147	27.1% (22.6%, 32.1%)	478	27.9% (25.3%, 30.7%)	
≥3	159	32.0% (27.0%, 37.4%)	498	30.6% (28.1%, 33.3%)	
Cancer Site					
Breast	140	24.3% (20.5%, 28.6%)	389	23.1% (20.6%, 25.9%)	0.10
Prostate	75	15.9% (12.5%, 19.9%)	241	15.1% (13.2%, 17.3%)	
Skin melanoma	39	7.1% (5.0%, 10.0%)	168	10.2% (8.6%, 12.1%)	
Multiple cancers	43	7.5% (5.4%, 10.4%)	186	10.5% (9.0%, 12.2%)	
Lung	14	2.6% (1.5%, 4.5%)	58	3.5% (2.62%, 4.6%)	
All other ^6^	221	42.6% (37.9%, 47.5%)	628	37.6% (35.0%, 40.2%)	
Family income level as % of FPL ^7^					
<200%	200	38.4% (33.7%, 43.4%)	457	27.9% (25.4%, 30.6%)	**<0.001**
200–399%	168	30.5% (26.2%, 35.1%)	556	33.9% (31.3%, 36.6%)	
≥400%	164	31.1% (26.7%, 35.8%)	657	38.2% (35.5%, 40.9%)	

Note: Bold font indicates statistical significance (*p* < 0.05). ^1^ Mean; %. ^2^ CI = Confidence Interval. ^3^ Chi-squared tests with Rao and Scott’s second-order correction for complex survey samples. ^4^ Other race/ethnicity include non-Hispanic Asian, American Indian or Alaska Native, any other group, or those who identify with other multiple races. ^5^ The number of comorbid conditions include self-reported arthritis, asthma, diabetes, COPD, coronary heart disease, hypertension, stroke, angina pectoris, and heart attack. ^6^ All other cancer sites include bladder, blood, bone, brain, cervical, colon, esophageal, gallbladder, larynx trachea, leukemia, liver, Uterine, lymphoma, melanoma, mouth, ovarian, pancreatic, rectal, stomach, throat, thyroid, head & neck, colorectal, and other. ^7^ FPL = federal poverty level (FPL).

**Table 2:** Weighted percentages of financial hardship associated with cancer, treatment, or lasting effects of treatment by Traditional Medicare and Medicare Advantage.

Outcome	Beneficiaries, % (95% CI)		Odds Ratio (95% CI) ^1,3^		
	Traditional Medicare	Medicare Advantage	Traditional Medicare	Medicare Advantage	*p*-Value ^2^
Material					
Problems paying medical bills					
	11.8% (8.6%, 25.8%)	10.3% (8.5%, 12.3%)	0.86 (0.58, 1.28)	1.04 (0.70, 1.55)	0.85
Unable to pay medical bills					
	6.2% (4.0%, 9.7%)	5.7% (4.5%, 7.1%)	1.14 (0.52, 2.47)	1.11 (0.50, 2.47)	0.79
Any material ^4^					
	11.8% (8.6%, 15.8%)	10.3% (8.5%, 12.3%)	1.17 (0.78, 1.74)	0.96 (0.64, 1.43)	0.84
Psychological					
Worry to pay due to sickness					
	38.2% (33.5%, 43.1%)	33.9% (31.3%, 36.6%)	0.83 (0.65, 1.05)	0.93 (0.73, 1.2)	0.59
Behavioral					
Delayed care due to cost					
	4.8% (2.9%, 7.8%)	3.0% (2.1%, 4.4%)	0.62 (0.33, 1.20)	0.70 (0.37, 1.31)	0.27
Forgone care due to cost					
	4.6% (2.6%, 8.1%)	3.3% (2.5%, 4.5%)	0.72 (0.36, 1.41)	0.90 (0.48, 1.67)	0.73
Delayed prescription medication					
	3.5% (2.2%, 5.6%)	3.6% (2.7%, 4.8%)	1.02 (0.57, 1.80)	1.11 (0.61, 2.03)	0.73
Forgone prescription medication					
	6.2% (3.8%, 10.0%)	4.3% (3.3%, 5.6%)	0.68 (0.38, 1.22)	0.78 (0.44, 1.40)	0.41
Delayed counseling due to cost					
	1.4% (0.7%, 2.8%)	0.7% (0.4%, 1.2%)	0.52 (0.21, 1.27)	0.44 (0.17, 1.11)	0.08
Forgone counseling due to cost					
	2.2% (1.1%, 4.2%)	1.0% (0.6%, 1.5%)	0.43 (0.19, 0.96)	0.39 (0.16, 0.92)	**0.03**
Any behavioral ^5^					
	12.0% (8.8%, 16.1%)	8.7% (7.2%, 10.4%)	1.44 (0.97, 2.14)	1.28 (0.86, 1.91)	0.22

Note: All the percentages are weighted percentages. Bold font indicates statistical significance (*p* < 0.05). ^1^ All regression controlled for survey year, age, sex, race, education, marital, region, number of comorbid conditions, and family income level as % of FPL. ^2^ *p*-values were derived using multivariable-adjusted logistic regression. ^3^ Presented with traditional Medicare as the reference group. ^4^ Any material financial hardship was defined as answering ‘yes’ to one or more of the individual material financial hardship measures including problems paying medical bills and/or being unable to pay medical bills. ^5^ Any behavioral financial hardship was defined as answering ‘yes’ to one or more of the adherence measures, including having delayed and/or forgone care due to cost, delayed and/or forgone prescription medication, and/or delayed and/or forgone prescription counseling due to cost.

**Table 3:** Weighted percentages of healthcare utilization associated with cancer, treatment, or lasting effects of treatment by Traditional Medicare and Medicare Advantage.

Outcome	Beneficiaries, % (95% CI)		Odds Ratio (95% CI) ^1,3^		
	Traditional Medicare	Medicare Advantage	Traditional Medicare	Medicare Advantage	*p*-Value ^2^
Any wellness visit ^4^					
	14.8% (11.6%, 18.7%)	16.8% (14.1%, 18.0%)	1.25 (0.63, 2.47)	1.40 (0.63, 3.14)	0.41
Any emergency service ^5^					
	39.9% (35.0%, 45.0%)	39.6% (36.7%, 42.5%)	1.01 (0.80, 1.29)	1.05 (0.83, 1.35)	0.67
Flu vaccination					
	70.1% (65.6%, 74.3%)	77.2% (75.0%, 79.4%)	1.44 (1.14, 1.83)	1.31 (1.03, 1.67)	**0.03**
Any hospitalization					
	20.0% (16.4%, 24.1%)	16.0% (14.1%, 18.0%)	0.76 (0.57, 1.02)	0.74 (0.56, 0.98)	**0.03**
Telehealth					
	33.4% (28.4%, 38.8%)	37.9% (35.2%, 40.6%)	0.82 (0.62, 1.07)	0.87 (0.67, 1.14)	0.33

Note: All the percentages were weighted percentages. Bold font indicates statistical significance (*p* < 0.05). ^1^ All regressions were controlled for survey year, age, sex, race, education, marital status, region, number of comorbid conditions, and family income level as % of FPL. ^2^ *p*-values were derived using multivariable-adjusted logistic regression. ^3^ Presented with traditional Medicare as the reference group. ^4^ This visit typically includes blood pressure, cholesterol, blood sugar checks, height and weight measurements, and vaccinations. ^5^ Any emergency services, including urgent care and ED visits.

## Data Availability

The publicly available dataset can be found at https://nhis.ipums.org/nhis/ (accessed on 10 November 2023).
